# Emerging tools in plant genome editing

**DOI:** 10.3389/fgeed.2025.1588089

**Published:** 2025-12-04

**Authors:** Shilpi Sharma, Naveen Kumar Saroha, Abhilasha Sehrawat, Guiliang Tang, Deepali Singh, Sachin Teotia

**Affiliations:** 1 Department of Biotechnology, Sharda University, Greater Noida, India; 2 Department of Biological Sciences, Michigan Technological University, Houghton, MI, United States; 3 School of Biotechnology, Gautam Buddha University, Greater Noida, India; 4 Department of Life Sciences, J.C. Bose University of Science and Technology, YMCA, Faridabad, India

**Keywords:** ARCUT, CRISPR-Cas, HACE, LEAPER, RESTORE, RESCUE, SPARDA, SATI

## Abstract

Plant genome editing has undergone a transformative shift with the advent of advanced molecular tools, offering unprecedented levels of precision, flexibility and efficiency in modifying genetic material. While classical site-directed nucleases such as ZFNs, TALENs and CRISPR-Cas9 have revolutionized genome engineering by enabling targeted mutagenesis and gene knockouts, the landscape is now rapidly evolving with the emergence of novel systems that go beyond the conventional double strand break (DSB)-mediated approaches. Advanced and recent tools include LEAPER, SATI, RESTORE, RESCUE, ARCUT, SPARDA, helicase-based approaches like HACE and Type IV-A CRISPR system, and transposon-based techniques like TATSI and piggyBac. These tools unlock previously inaccessible avenues of genome and transcriptome modulation. Some of these technologies allow DSB-free editing of DNA, precise base substitutions and RNA editing without altering the genomic DNA, a significant advancement for regulatory approval and for species with complex genomes or limited regeneration capacity. While LEAPER, RESCUE and RESTORE are the new advents in the RNA editing tool, SATI allows DSB-free approach for DNA editing, ARCUT offers less off-target and cleaner DNA repairs and Type IV-A CRISPR system induces gene silencing rather than editing. The transposon-based approaches include TATSI, piggyBac and TnpB, and helicases are used in HACE and Type IV-A CRISPR system. The prokaryotic Argonaute protein is used in SPARDA tool as an endonuclease to edit DNA. The transient and reversible nature of RNA editing tools such as RESTORE and LEAPER introduces a new layer of epigenetics-like control in plant systems, which could be harnessed for tissue-specific and environmentally-responsive trait expression. Simultaneously, innovations like ARCUT and SPARDA utilize chemically-guided editing, minimizing reliance on biological nucleases and reducing off-target risks. Their modularity and programmability are enabling gene function studies, synthetic pathway designs and targeted trait stacking. These advances represent a novel synthesis of genome engineering and systems biology, positioning plant genome editing not just as a tool of modification but as a platform for designing adaptive and intelligent crops, tailored to future environmental and nutritional challenges. Although, many of these recent tools remain to be applied on plant systems, they are proven to be effective elsewhere and hold a great potential to be effective in creating climate-resilient crops.

## Introduction

Introgression of desirable traits through conventional breeding is time-consuming and often restrained by genetic bottlenecks (Atia et al., 2024). Recent advances in genome editing technologies have brought revolutionary changes in the field of biotechnology. The tools have eased targeted and precise modifications of the genetic material in various organisms, including plants. Genome editing involves precise modifications of DNA sequences at targeted regions in the form of deletions, insertions, substitutions, base-editing, homology-based substitutions, and modulation of gene expression to introduce desirable traits in crop plants. Genome editing has gained unprecedented importance in agricultural research and breeding programs, addressing global challenges of creating climate-resilient crops ensuring food security and environmental sustainability. Genome editing aims at inducing DNA alterations with minimal off-target effects. In addition, genome modifications which are of transient and reversible nature are desirable for trait management and fulfil the regulatory compliance for commercial release.

The first genome editing methods utilized programmable nucleases including Zinc Finger Nucleases (ZFNs) and Transcription Activator-Like Effector Nucleases (TALENs) to generate targeted double-strand breaks (DSBs) at the desired genomic loci (Gupta and Musunuru, 2014). Subsequently, the cellular repair mechanisms of non-homologous end joining (NHEJ) and homology-directed repair (HDR) get activated to repair these DSBs. Following that, the CRISPR-Cas system gained precedence over older tools because of their large size and complexity, which limited their efficiency (O'Connell et al., 2014). The CRISPR-Cas system emerged from the bacterial immune systems and gained popularity as the prevalent genome editing tool because of its user-friendly nature, accuracy and versatility (Doudna and Charpentier, 2014). The CRISPR-Cas9 system uses a guide RNA (gRNA) that guides the Cas9 nuclease to target DNA sequence to create a DSB. The subsequent cellular repairs executed through NHEJ or HDR procedures execute targeted modifications that create deletions, insertions and substitutions (Hsu et al., 2014). ZFNs demand labor-intensive protein engineering for each target, limiting their scalability. Like ZFNs, TALENs also offer higher specificity due to longer DNA recognition sequences but are technically cumbersome and time-consuming to construct as they require complex protein engineering for each new target site. Their large size also complicates delivery, particularly in plant systems, with limited transformation capacity. ZFNs are constrained by their context-dependent DNA-binding, where individual zinc finger domains influence the binding of adjacent ones, making target site design non-trivial. The size and complexity issues pertaining to ZFNs/TALENs are done away in the CRISPR-Cas system. Despite their transformative impact on genome engineering, CRISPR-Cas9 is susceptible to off-target mutations due to tolerance to mismatches between the gRNA and genomic DNA, particularly in genomic regions with high sequence similarity. Its dependence on the presence of a protospacer adjacent motif (PAM) sequence also restricts target site availability. In addition, CRISPR often induces DSBs, which can lead to unpredictable repair outcomes and genomic instability, especially in species with inefficient cellular repair pathways. The designing, applications and limitations of meganucleases, ZFNs, TALENS and CRISPR-based tools have been discussed extensively before (Epinat et al., 2003; Carroll, 2011; Silva et al., 2011; Sun and Zhao, 2013; Cardi et al., 2023).

Abiotic stresses such as drought, salinity, and extreme temperatures adversely impact crop yields worldwide. Genome editing technologies have been used to modify key regulatory genes underlying these stress response pathways leading to enhanced tolerance to these stressors (Kumar et al., 2023). For example, genes have been edited in rice by CRISPR-Cas9 leading to increased yield under drought conditions (Sun et al., 2017). CRISPR-based approaches have been used to modify resistance genes, enhancing plant immunity against fungal, bacterial, and viral pathogens (Mahfouz et al., 2016). For instance, CRISPR-Cas9-mediated editing of the *eIF4E* gene in tomatoes has conferred resistance to potyviruses (Yoon et al., 2020). Additionally, transposon-based genome editing has been utilized to create resistance traits in crop plants without disrupting native genomic integrity (Liu et al., 2024). Precise modifications in genes governing metabolic pathways have enabled the development of crops with increased vitamin and mineral content, improving dietary content (Zhu et al., 2020).

Despite the success of CRISPR-Cas9 and its derivatives, recent advancements have introduced new genome editing technologies that offer even greater precision, efficiency, and regulatory advantages. Tools such as LEAPER, SATI, RESTORE and RESCUE provide alternative potential approaches for modifying plant genomes without inducing DSBs, thereby reducing the risks of off-target effects and genomic instability (Gorucu Yilmaz, 2021). In this review, we are discussing all these newly developed tools which can be potentially used in plant genome editing (Table 1).

**TABLE 1:** Different features of emerging genome editing tools.

Technology	Salient feature	Target	Cutting tool	Guiding tool	Mode of action	Off-target	Potential application in plants	References
CRISPR	Efficient, specific, easy, allows multiplexing	DNA	Cas nuclease	Guide RNA (gRNA)	gRNA-guided Cas9 cuts DNA near PAM site that undergoes repair (NHEJ or HDR)	Depends on gRNA design	Precise editing of targeted genes to enhance traits, impart-climate resilience, disease resistance in plants	Doudna and Charpentier (2014)
LEAPER	Precise, easy, transient, reversible	RNA	ADAR1 enzymes	Long antisense ADAR-recruiting RNAs (arRNAs)	Utilizes arRNA. and provide A-to-I conversion and recruiting the endogenous ADAR1 enzymes	Minimum off-target	Achieve desirable agronomic traits, creating virus-resistant plants by editing the RNA of viral genes	Qu et al. (2019)
RESTORE	Targets mRNA binding with a programmable specific domain	RNA	ADAR (adenosine deaminase acting on RNA)	Chemically modified oligonucleotide gRNAs	Point mutations (A-to-I) in the transcripts related to the trait	Minimum off-target	Precision breeding, Gene silencing or activation	Merkle et al. (2019)
RESCUE	C-to-U deamination activity	RNA	ADAR	gRNAs	C-to-U conversion in RNA	Minimum off-target	Gene expression modulation	Abudayyeh et al. (2019)
ARCUT	Use of synthetic oligonucleotides	DNA	Cerium (IV) ethylenediamine-N,N,N′,N′-tetraacetic acid (Ce(IV)/EDTA)—in combination with peptide nucleic acid sequences	Pseudo-Complementary Peptide Nucleic Acid (pcPNA)	Supports homologous recombination	Minimum off-target	Imparting disease resistance, Environmental stress tolerance	Shigi and Komiyama (2010)
SATI	Uses DNA repair pathways HDR and NHEJ, DNA knock-in target flexibility, DSB-free	DNA	Homology directed mismatch repair machinery	Single-stranded oligonucleotides (ssODNs)	Adds new segment to the genome without deleting the previous segment	Not determined	Induced mutagenesis	Suzuki et al. (2019)
TATSI	Combines CRISPR-Cas9 with the transposon system for sequence-specific DNA integration	DNA	Cas9 with transposase	gRNA	Site-specific integration	Less common	Specific integration for crop improvement	Liu et al. (2024)
SPARDA (Short Prokaryotic Argonaute-DNase and RNase Associated)	DNA or RNA degradation with the help of an associated effector nuclease	RNA or DNA	pAgo nuclease	gRNA or DNA guide	Cleaves DNA or RNA at the target site and enables targeted degradation or editing	Low as compared to CRISPR-Cas9	Precise genome editing	Prostova et al. (2024)
PiggyBac Transposon System	Scarless integration with large cargo insertion	DNA	PiggyBac Transposase with Cas9	gRNA	Site-specific integration	Less prone to off-target effects	Delivery of transgene	Li et al. (2013), Nishizawa-Yokoi and Toki (2023)
Tiny TnpB (Compact Genome Scissors)	Easier delivery and higher efficiency due to smaller size of TnpB	DNA	TnpB nuclease	gRNA	Introduces double-strand breaks (DSBs) and initiate DNA repair mechanisms	Potential off-target breaks	Efficient genome editing	Li et al. (2024), Karmakar et al. (2024)
HACE	Uses Helicase-deaminase directed by CRISPR-Cas9 or ZFNs or TALENs	DNA	Cas9 nuclease or ZFNs or TALENs together with helicase	gRNA	Helicase-deaminase continuously unwinds DNA and changes nucleotides to create mutations	Low as compared to CRISPR-Cas	Precise, long range and targeted editing of genes	Chen et al. (2024)
Type IV-A CRISPR system	Uses helicase enzyme to target DNA along with other Cas proteins to induce transient and reversible gene silencing	DNA	Csf proteins together with DinG helicase	gRNA	Silences gene expression without cutting DNA	Low as compared to CRISPR-Cas	Identification and regulation of gene expression of important traits	Cui et al. (2023)

RNA editing systems such as LEAPER, RESCUE and RESTORE allow temporary changes to the genetic material without affecting the genome’s structure, making them prospective tools for creating crops that can respond to environmental stresses (Qu et al., 2019; Aquino-Jarquin, 2020). Transposon-based systems like TATSI and piggyBac allow gene insertions, without exogenous DNA integration. Such an approach will enable modified crops to comply with the regulatory norms (Liu et al., 2024). TATSI and piggyBac offer a significant advantage over conventional integration approaches as they avoid low frequency and random insertion with unintended mutations in the genome. Notably, piggyBac enables footprint-free transposition ensuring cleaner edits. Furthermore, technologies like HACE and SPARDA enhance genome accessibility and editing efficiency by exploiting helicase and Argonaute-based mechanisms, respectively, further broadening the scope of plant biotechnology (Chen et al., 2024; Prostova et al., 2024). While HACE enhances accessibility via helicase activity, SPARDA is a bi-partite immune system consisting of a short prokaryotic Argonaute (pAgo) protein and co-encodes nuclease, with indiscriminate cleavage activity. The study shows that the DNA and RNA effector nuclease (DREN), is activated only upon the binding of gRNA to the target DNA (RNA-guided DNA recognition). SATI is a DSB-free inducing editing tool, useful to negate unpredictable consequences of NHEJ repairs.

## CRISPR-Cas system

The CRISPR-Cas system functions as a two-component system comprising the gRNA and the Cas9 enzyme, which collaborate to allow the precise editing of genes in various organisms (Makarova et al., 2011; Makarova and Koonin, 2015). The synthetic gRNA consists of two parts- crRNA and tracrRNA. The crRNA harbors a sequence complementary to the target DNA sequence within the genome. It guides the Cas9 enzyme to the targeted site that needs a cut. The tracrRNA aids in stabilizing the interaction between the crRNA and the Cas9 enzyme (Doudna and Charpentier, 2014). Cas9, an RNA-guided DNA endonuclease enzyme, works for bacterial immunity to protect against foreign DNA such as bacteriophage or plasmid DNA, using the memory of their first attack (Heler et al., 2015). At the target locus, Cas9 initiates a DSB after identifying the PAM sequence (Gleditzsch et al., 2019). The CRISPR-Cas9 system is the type II CRISPR system, which depends on a single Cas protein from the *Streptococcus pyogenes* (SpCas9). The cellular repair mechanisms either, leads to gene disruption in case errors are introduced (NHEJ) or involves integration of new genetic material, in case, the cell receives a template to repair the break (HDR) (Doudna and Charpentier, 2014). This allows us to use CRISPR-Cas9 for knocking out genes, correcting mutations, base editing or inserting new gene fragments in the genomes of various organisms. It thus opens wide-ranging applications in genetic research, medicine, and agriculture.

There are six known types of CRISPR-Cas and at least 29 subtypes (Makarova and Koonin, 2015; Makarova et al., 2011; Makarova et al., 2020). CRISPR–Cas systems have been classified into 2 major classes based on their evolutionary relationships (Makarova et al., 2020). The class 1 systems have effector modules composed of multiple Cas proteins, some of which form crRNA-binding complexes (such as the Cascade complex in type I systems) that, with contributions from additional Cas proteins, mediate pre-crRNA processing and interference. Whereas the class II systems encompass a single, multidomain crRNA-binding protein (such as Cas9 in type II systems) that combines all activities required for interference and, in some variants, also those involved in pre-crRNA processing (Makarova et al., 2020). The prokaryotic immune system consists of two proteins known as Cas1 and Cas2. While the endonuclease Cas1 enables the generation of dsDNA fragments, Cas2 controls spacer acquisition in CRISPR-Cas adaptive immunity and stabilizes the complex with Cas1 (Nunez et al., 2014). Another Cas protein, Cas3, participates in the next step of CRISPR intervention and is necessary for the CRISPR-Cas system’s phage defence (He et al., 2020; Liu et al., 2020). To protect bacterial cell from viral infection, another Cas protein, Cas4, a 5′ to 3′ DNA exonuclease with an Iron-Sulfur Cluster, aids in the creation of memories for invasive viral components (Zhang et al., 2012). Cas4 is required for better pre-spacer processing prior to Cas1-Cas2-mediated spacer acquisition (Lee et al., 2018). When Cas4 is not present, the bacteria produce an invasion memory but are unable to retain it. Main reason for Cas4 not being able to retain memory is the presence of a short DNA sequence made up of a limited number of base pairs that serves to pair with proteins recognizing PAM (Kieper et al., 2018). The Cas5 and Cas6 are the other proteins of the CRISPR systems. Cas5 acts as a substitute for Cas6 when it is catalytically active. Cas6 and 7 belong to the repeat-associated mysterious proteins (RAMP) superfamily and process pre-crRNA transcripts by sequence- or structure-specific RNAse activity (Makarova et al., 2011; 2020). Cas12 (Cpf1/Cas12a) and Cas13 are the most studied Cas proteins after Cas9 (Swarts and Jinek, 2018). With its CRISPR array processing efficiency, Cas13 is ideal for applications involving several targets. Most importantly, it is widely used in clinical applications and biotechnology. With Cas13 (SHERLOCK), targeted DNA or RNA sequences can be precisely detected (Kellner et al., 2020). A number of reviews in the past have already highlighted the classification of CRISPR-Cas systems (Makarova and Koonin, 2015; 2018; Xu and Li, 2020; Koonin et al., 2017).

## RNA editing tools

### RNA Editing for Programmable A to I Replacement (REPAIR)

Cas13 has an ability to bind to target RNA and trigger collateral cleavage of nearby RNA forms and this property forms the basis of highly sensitive diagnostic platforms such as SHERLOCK. SHERLOCK has been successfully employed for rapid, point-of-care detection of viral pathogens like SARS-CoV-2, Zika, and Dengue. However, due to its indiscriminate RNA cleavage after activation, Cas13 is not inherently suitable for precise RNA editing. For targeted RNA editing, technology like REPAIR is more appropriate. This system pairs catalytically inactive Cas13 (dCas13) with engineered deaminases to mediate site-specific base conversions (A-to-I) on RNA without collateral damage. The RNA editing system was reported by fusing dCas13 to adenosine deaminase domain of ADAR2 (adenosine deaminase acting on RNA type 2) to direct adenosine-to-inosine (A-to-I) edits in RNAs of mammalian cells. However, the system comes with a sizeable number of A-to-I off-target events. As Cas13 has no bias for PAM like motifs, REPAIR system can be used to target any adenosine in the transcriptome. The A-to-I conversions are not based upon endogenous cellular repairs such as base-excision or mismatch repair (Cox et al., 2017), limiting the application of this tool in genome editing.

### Leveraging Endogenous ADAR for Programmable Editing of RNA (LEAPER)

**FIGURE 1:**
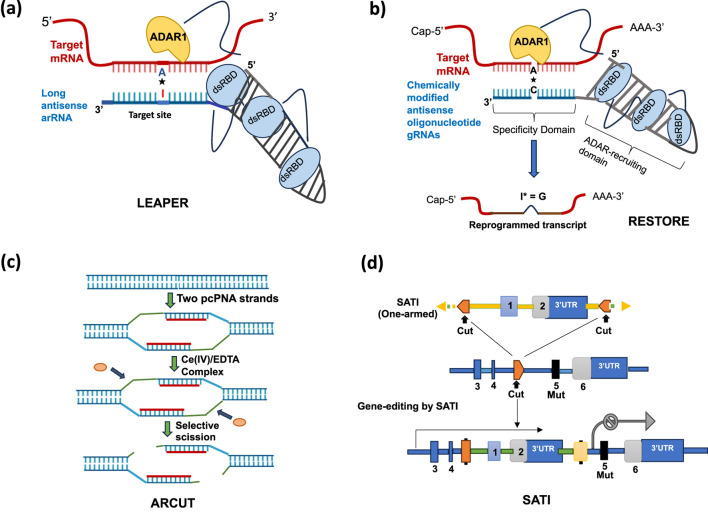
**(a)** LEAPER editing technique which utilizes the endogenous activity of Adenosine ADARs to catalyze the modification of adenosine (A) to inosine (I) in double-stranded RNA. The LEAPER system is based on three components: the target RNA with the adenosine to be edited, specifically designed antisense ADAR-recruiting RNAs (arRNAs) corresponding to the target region, and the endogenous ADARs that mediate the A-to-I modification with or without the Cas13 protein. The arRNA binds onto the target RNA and forms a structure that ADAR enzymes can recognize and deaminate the adenosine (A) at the target region to inosine (I). **(b)** RESTORE RNA editing technique, which alters specific mRNA transcripts using endogenous ADAR through antisense oligonucleotides (ASO). One of the two segments of an antisense ASO targets mRNA binding-determining programmable specificity domain and the other segment binds with the ADAR-recruiting domain which guides endogenous ADARs to the ASO:mRNA hybrid. ADARs preferentially deaminate A to I within imperfect dsRNA helices, particularly when the target ‘A’ is positioned in a bulged or mismatched region of the helix. dsRBD stands for “double-stranded RNA binding domain,” a protein motif found in ADAR enzymes that allows them to specifically bind to dsRNA molecules. dsRBD acts as the key component that guides the ADAR enzyme to the desired location on the RNA to perform editing. **(c)** Site selective scission of double stranded DNA by ARCUT. “pcPNA” stands for “pseudo-complementary Peptide Nucleic Acid.” This system employs a chemically engineered molecular scissors—cerium (IV) ethylenediamine-N,N,N′,N′-tetraacetic acid (Ce(IV)/EDTA)—in combination with pcPNA sequences. The site-selective scission proceeds via hydrolysis of targeted phosphodiester linkages, so that the resultant scission fragments can be easily ligated with other fragments by using DNA ligase. **(d)** SATI gene-editing process using one homology arm for the targeted integration of a donor segment into an intron. SATI uses alignment of ssODNs with complementary sequences in the target DNA, to facilitate the precise insertion or replacement of genetic material without involving DSBs.

Following REPAIR system, LEAPER technology has become an effective tool for RNA editing. This technique was discovered by a team of researchers at Peking University, China. LEAPER induces modifications of RNA in a transient and reversible fashion. The process utilizes the endogenous activity of Adenosine Deaminases Acting on RNA (ADARs) to catalyze the modification of adenosine (A) to inosine (I) in double-stranded RNA (Figure 1a). The LEAPER system is based on three components: the target RNA with the adenosine to be edited, specifically designed supplemental RNA oligonucleotides, ADAR-recruiting RNAs (arRNAs) corresponding to the target region, and the endogenous ADARs that mediate the A-to-I modification with or without the Cas13 protein (Yi et al., 2022; Aquino-Jarquin, 2020). The arRNAs are designed to base pair with target RNA sequences so that the site-specific A-to-I editing can be achieved by recruiting endogenous ADARs (Figure 1a). After being introduced into the plant cell, the arRNA binds onto the target RNA and forms a structure that ADAR enzymes can recognize. The ADAR enzymes deaminate the adenosine at the target region to inosine (Qu et al., 2019; Aquino-Jarquin, 2020). Since inosine replaces guanosine during the process of translation, it changes the codon in question and those that follow (Savva et al., 2012). This enables the system to achieve editing efficiency of up to 80% with low global off-target impacts and non-target adenosines editing within the target site (Qu et al., 2019). For plant systems, LEAPER provides an effective alternative for transient RNA editing because it does not rely on additional components.

The LEAPER technology has several benefits in comparison to customary genome editing, especially in agricultural contexts. One of these benefits is safety and transience. RNA amendments are reversible and temporary, relieving worries involving permanent DNA alterations, including unintended genetic effects and regulatory challenges on genetically modified organisms (GMOs). Additionally, LEAPER does not need the introduction of foreign proteins, using only synthetic RNA and native enzymes, thereby minimizing the chance of immunogenicity and facilitating delivery in plants. Moreover, LEAPER’s high specificity through the design of arRNAs with high sequence complementarity to the target region, guarantees accurate editing with minimal off-target effects—a critical consideration for agricultural use. A major limitation of LEAPER in plant systems is the potential off-target RNA editing. arRNAs can bind unintended RNA transcripts with partial complementarity, especially given the complexity and isoform diversity of plant transcriptomes. Such off-target binding can lead to widespread and unpredictable A-to-I conversions, which may affect untranslated regions, splicing elements, microRNA binding sites, or regulatory RNAs. These unintended edits can disrupt gene expression, alter protein function, or interfere with RNA stability, leading to aberrant plant phenotypes or stress responses. Additionally, epitranscriptomic instability arising from excessive inosine accumulation could further affect transcript localization and translation efficiency in plant cells. To mitigate these risks, several strategies have been developed to improve arRNA design and minimize off-target activity. Rational design approaches focus on minimizing arRNA complementarity to non-target transcripts through transcriptome-wide specificity screening. Shortening arRNA length to approximately 70–100 nucleotides, reduces promiscuous binding while maintaining on-target affinity. Introducing intentional mismatches or structural bulges near non-target adenosines within the guide can further restrict undesired editing. Computational tools, such as LEAPER-2.0, help assess potential off-targets and optimize arRNA sequences (Yi et al., 2023). In addition, RNA secondary structure modeling, using tools like RNAfold, can guide the selection of target sites in structurally accessible, single-stranded regions that are less prone to non-specific ADAR recruitment. Chemical modifications, such as 2′-O-methylation or phosphorothioate linkages, enhance arRNA stability and fine-tune binding specificity. Moreover, engineering arRNAs with subcellular localization signals, can help to spatially confine the editing activity, further limiting transcriptome-wide exposure.

LEAPER technology certainly has an array of advantages; however, its application to plants still poses several tricky issues. Out of the many problems that embody it, one primary challenge could be its dependence on endogenous ADAR expression. There are a few eukaryotes that have ADAR-like enzymes, but there is no information regarding their presence and functioning in plants. To perform effective RNA editing, plants may need to be modified to express functional ADARs. Additionally, unintended off-target editing remains a concern, particularly in plants with complex genomes. The creation and verification of improved arRNA designs, seems to be one of the potential solutions to alleviate these risks. Another challenge relates to the constrictions surrounding the delivery of arRNAs to specific plant tissues or compartments. Tools like nanoparticle delivery systems or agroinfiltration or even viral vectors will need to be tweaked for making arRNAs delivery in plants. Agrobacterium-mediated transient expression remains a commonly used method for introducing arRNA-expressing DNA constructs into dicotyledonous plants, such as *Nicotiana benthamiana*. This method allows systemic delivery and high expression levels under plant promoters; however, it is largely restricted to dicots, may trigger immune responses, and involves the use of DNA vectors, which raises regulatory concerns in non-GMO contexts. Viral vectors, including tobacco rattle virus (TRV) and potato virus X (PVX), are also popular for delivering RNA in plants. These systems can allow rapid and systemic spread of the arRNA throughout the plant without stable genome integration. While viral vectors offer high efficiency and the potential for multiplexed arRNA delivery, they are limited by their cargo size, host range, and potential interference with host gene expression or metabolism. Nanoparticle-based delivery methods offer a promising DNA-free alternative for arRNA application. These nanoparticles can be applied topically to leaves and facilitate passive diffusion or endocytosis into plant cells. Their major advantages include non-transgenic delivery, species-independence, and scalability for agricultural use. However, challenges remain regarding their limited tissue penetration, uneven distribution, environmental persistence, and regulatory scrutiny. Polyethylene glycol (PEG)-mediated delivery into protoplasts represents another precise approach, allowing direct transfection of RNA into isolated plant cells. While useful for *in vitro* validation and mechanistic studies, this technique is labor-intensive, species-dependent, and impractical for full plant transformation due to regeneration limitations. Biolistic particle bombardment (gene gun) provides a physical method to deliver arRNAs or their DNA templates by coating them onto gold or tungsten particles and shooting them into plant tissues. Though applicable across diverse species including monocots, this method often results in localized and transient expression and may cause tissue damage, limiting its scalability. Electroporation of protoplasts is another option, permitting rapid transfection of RNA, but like PEG-mediated delivery, it is restricted to cell culture systems and unsuitable for whole plant application. In conclusion, while each delivery method has its own trade-offs, viral vectors and nanoparticle-mediated delivery currently offer the most practical routes for delivering arRNAs into whole plants in a non-integrative, potentially field-deployable manner. PEG and biolistic methods, although valuable in research settings, face scalability and tissue regeneration challenges. Continued optimization and development of efficient, safe, and regulatory-compliant delivery systems will be critical to the future success of LEAPER-mediated RNA editing in plant biotechnology.

LEAPER has been primarily validated in mammalian cells, including human cell lines and animal models, where endogenous ADAR expression is sufficient to drive the desired A-to-I editing. However, its applicability in plants remains largely unexplored, largely due to the lack of functional characterization of plant ADAR-like enzymes, which are either absent or functionally distinct from their mammalian counterparts. Despite these constraints, LEAPER holds considerable potential as a future tool for plant biotechnology. If functional equivalents of ADAR or analogous editing enzymes can be identified or engineered in plants, LEAPER could offer transient, programmable RNA editing without altering the genome, thus potentially bypassing regulatory hurdles associated with DNA editing. Such a system could be harnessed for reversible gene regulation, stress response modulation, or fine-tuning developmental pathways.

### Recruiting Endogenous ADAR to Specific Transcripts for Oligonucleotide-mediated RNA Editing (RESTORE)

A programmable system called RESTORE is employed for site-directed RNA editing. Before RESTORE, RNA editing has relied upon either overexpression of exogenous RNA editing enzymes or endogenous human ADAR enzymes. RESTORE uses antisense oligonucleotides (ASO) to deploy endogenous human ADARs to edit endogenous transcripts in a regulated manner. The system consists of two segments of an antisense ASO that have been chemically altered and engineered, one is a target mRNA binding-determining programmable specificity domain and another one is ADAR recruiting domain which guides endogenous ADARs to the ASO:mRNA hybrid (Merkle et al., 2019). ADARs preferentially deaminate A-to-I within imperfect dsRNA helices, particularly when the target adenosine is positioned in a bulged or mismatched region of the helix. RESTORE exploits this preference by designing ASOs that are complementary to the target mRNA sequence but deliberately introduce a mismatch or bulge at the adenosine to be edited. This bulged ‘A’ within an otherwise stable dsRNA duplex creates a structure that closely resembles the natural ADAR substrate effectively recruiting endogenous ADAR enzymes to the site without needing any engineered protein component. The surrounding double-stranded region ensures stable hybridization, while the specific configuration around the target adenosine (A) enhances ADAR’s affinity and catalytic activity. By mimicking these structural and spatial requirements, the ASO:mRNA duplex becomes a highly efficient and selective substrate for A-to-I editing, ensuring targeted modification with minimal off-target effects. At the transitional level, editing with RESTORE is most effective. With editing results ranging from 75% to 85%, scientists may fix mutations that are clinically significant with minimum off-target editing effect (Aquino-Jarquin, 2020). Endogenous ADAR, or chemically optimized ASOs, are used in this technique.

RESTORE achieves minimal off-target through rational ASO design, chemical modifications and transcript-specific targeting. ASOs are carefully designed to form highly specific short duplexes with the target RNA, so that the target adenosine is placed in the bulged or mismatched configuration within the helix so that the editing occurs only at the specific adenosine which is the preferred substrate for endogenous ADARs. Chemical modifications such as 2′-O-methyl ribose and phosphorothioate linkages enhance ASO stability while reducing non-specific interactions. By leveraging endogenous ADAR rather than exogenous editing enzymes, RESTORE limits off-target activity and avoids unwanted cellular stress. Moreover, RESTORE targets RNA rather than DNA, allowing for isoform specific and reversible editing, which further minimizes the risk of permanent genomic alterations or unintended transcriptome-wide effects.

Without chemical modifications, ASOs are highly vulnerable to degradation by nucleases present in the cellular environment, significantly, reducing their half-life and rendering them ineffective for *in vivo* applications. Common modifications such as phosphorothioate (PS) backbones and 2′-O-methyl or 2′-O-methoxyethyl (2′-MOE) ribose substitutions are frequently employed to increase nuclease resistance and improve pharmacokinetics. These modifications also reduce immune activation, which is especially important in therapeutic contexts to avoid unintended inflammatory responses. Additionally, careful chemical tuning allows the ASOs to form optimal secondary structures—like hairpins or loops—that enhance ADAR binding and editing efficiency while minimizing off-target effects. If such chemical modifications are omitted, several adverse consequences can occur. First, unmodified ASOs are rapidly degraded, resulting in poor bioavailability and transient expression, severely compromising RNA editing efficiency. Second, their structural instability may lead to misfolding or suboptimal hybridization with the target RNA, reducing on-target editing rates and potentially increasing nonspecific binding. Third, unmodified oligonucleotides may inadvertently trigger innate immune responses through recognition by Toll-like receptors, leading to cytotoxic effects or systemic inflammation. Therefore, chemical modification is not merely an enhancement but a prerequisite for the RESTORE platform’s effectiveness and safety. Strategic design incorporating both backbone and sugar modifications ensures that ASOs maintain their integrity, specificity, and capacity to recruit endogenous ADAR enzymes efficiently for precise RNA editing.

While LEAPER uses long, circular antisense RNAs expressed within the cell, RESTORE uses chemically synthesized oligonucleotide gRNAs that require complex modifications for stability and delivery. LEAPER relies more on the stable expression within the cell, while RESTORE relies on external delivery of specially designed oligonucleotide guides (Figure 1b).

### RNA Editing for Specific C to U Exchange (RESCUE)

Another base-editing approach that directly convert cytidine to uridine in RNA by utilizing engineered deaminases is RESCUE (Cox et al., 2017; Fry et al., 2020). It was created by engineering the enzyme ADAR2 into a cytidine-to-uridine (C-to-U) deaminase from previously A-to-I RNA editing of REPAIR and LEAPER systems (Abudayyeh et al., 2019). RESCUE technology utilizes an engineered cytidine deaminase enzyme, fused with a sequence-targeting domain like dCas13 or an RNA-binding protein, to introduce sequence-specific C-to-U conversions. Guide RNAs that come along with the targeting domain are responsible for specificity through hybridization with complementary sequences of the target RNA (Ichinose and Sugita, 2016; Wang et al., 2016). The key to RESCUE’s editing capability lies in the use of an engineered ADAR2 deaminase domain. While ADAR2 naturally catalyzes A-to-I editing in double-stranded RNA, the engineered enzyme, created with specific point mutations (E488Q, R455K and T375G) in the catalytic domain, has an altered substrate specificity, thereby enabling the enzyme to recognize and deaminate cytidine instead of adenosine. Positively charged amino acid residues are introduced on the deaminase surface to increase electrostatic interactions with the RNA backbone, improving binding affinity and orientation and maintaining a stable RNA-protein complex. The deaminase domain is fused to the catalytically inactive dCas13 which guides the complex to a specific RNA region, narrowing the editing window to usually 4-6 nucleotides, thereby ensuring that only the intended cytidine is exposed to the deaminase active site. This engineered deaminase then catalyzes the hydrolytic removal of the amino group from the cytidine base, converting it into uridine (C→U). The reaction occurs within a short editing window, typically 4–7 nucleotides upstream of the gRNA’s protospacer-flanking sequence. The resultant U is then interpreted by the translation machinery as a thymidine analog, leading to a codon change that can restore or modify protein function. Cytidine deaminase activity is critical for C-to-U conversion: natural cytidine deaminases such as APOBEC1 and CDA catalyze C-to-U conversions through hydrolytic deamination of the amine group at position 4 of the cytosine ring (Abudayyeh et al., 2019). However, these cytidine deaminases often have broader off-target effects and act on both DNA and RNA. RESCUE utilizes modified and programmable ADAR2 deaminase to act as a highly selective RNA-targeted cytidine deaminase. This allows transcript-level modifications that are transient and reversible.

RESCUE minimizes off-target RNA editing by combining a high-fidelity RNA binding module of dCas13b with an engineered, position-specific cytidine deaminase, and by tight structural and functional constraints through gRNA design. These factors collectively ensure that editing occurs only in the intended transcripts with precise cytidine targeting, avoiding cleavage in RNAs with partial complementarity.

RESCUE provides a transient and accurate option for correcting deleterious mutations at the RNA level and its reversibility eliminates risk related to off-target DNA edits. RESCUE carries out highly precise RNA editing without causing permanent genetic changes, which makes it a safer option compared to DNA editing methods (Abudayyeh et al., 2019; Booth et al., 2023). Transient editing ensures that any off-target effect is temporary and inheritable, reducing long term toxicity. However, for chronic or progressive conditions, the therapeutic benefit of a single editing event may not be sufficient. Since the edited RNA is short-lived, the treatment must be repeated or maintained through sustained delivery to preserve therapeutic efficacy. Therefore, a reliable and efficient delivery system can ensure consistent expression. Moreover, chemical modifications of gRNAs or antisense oligonucleotides can enhance stability and reduce degradation, extending their activity *in vivo*. Several strategies such as use of self-replicating RNA systems, tissue specific or inducible promoters and advanced encapsulation is adopted to prolong intracellular persistence. Overall, the transient editing provided by RESCUE can be a powerful tool in both therapeutic and agricultural settings, provided it is coupled with robust delivery mechanisms and dosing strategies to ensure sustained benefits wherever required.

Unlike a DNA editing tool, RESCUE works solely at the RNA level, allowing its application in various cell types and organisms without the limitations of DNA delivery (Abudayyeh et al., 2019; Booth et al., 2023). By preventing DNA DSBs, RESCUE minimizes the threats of genome instability and off-target mutagenesis. Although RESCUE is designed with high specificity, low-frequency off-target editing can still occur, especially in transcripts with partial complementarity. However, such risks are relatively low compared to permanent genome editing tools and are transient due to RNA turnover. Still, careful gRNA design, transcriptome-wide off-target prediction, and validation through RNA-seq are essential to minimize these unintended consequences. Improvements to RESCUE technology, such as integration into CRISPR-Cas systems for improved targeting, may widen its applicability.

Both DNA base editing and RESCUE aim for precise nucleotide modifications without inducing DSBs, their molecular targets and therapeutic implications differ significantly. RESCUE edits RNA, making it transient and reversible which is advantageous for temporary gene regulation where long term modification is risky, whereas DNA base editing results in permanent and heritable changes in the genome. This is ideal for one-time corrections of deleterious mutations. DNA base editors are highly efficient in dividing cells while RESCUE functions independently of cell division. DNA base editors are larger proteins making them difficult to deliver, RESCUE can be packaged into smaller vectors or delivered as nanoparticles. LEAPER and RESTORE systems come with the multiplexing option. The difference lies in the usage of their RNA editing strategy such as dCAS13-ADAR2 fusion for RESCUE, ASO-ADAR fusion for RESTORE and arRNAs for LEAPER (Aquino-Jarquin, 2020). The editing efficiencies of RESTORE and LEAPER are comparable as they use longer gRNAs (60–90 bp for RESTORE and 110–150 bp for LEAPER). The RNA editing efficiency of RESCUE is almost the half as it uses a shorter gRNA of about 30 bp. RESTORE relies upon ASOs-mediated transfection while LEAPER uses plasmid or lentiviral vector, and RESCUE uses plasmid transfection.

## DNA editing tools

### Artificial Restriction Cutter Technology (ARCUT)

ARCUT technology is a sophisticated genome editing tool designed to achieve unparalleled precision and specificity (Shigi and Komiyama, 2010; Komiyama, 2013). It employs artificial restriction enzymes to target and cleave DNA at specific sites. This system employs a chemically engineered molecular scissor—cerium (IV) ethylenediamine-N,N,N′,N′-tetraacetic acid (Ce(IV)/EDTA)—in combination with peptide nucleic acid (PNA) sequences (Hanvey et al., 1992). The site-selective scission proceeds via hydrolysis of targeted phosphodiester linkages, so that the resultant scission fragments can be easily ligated with other fragments by using DNA ligase (Yamamoto et al., 2004). Cleavage occurs via hydrolysis of the phosphodiester bond producing clean and staggered DNA ends without generating oxidative damage to bases or sugars that are readily processed for homologous recombination. In contrast, Cas9-induced blunt DSBs can be rapidly sealed by the dominant repair mechanism, NHEJ. The Ce(IV)/EDTA complex achieves sequence-specific DNA cleavage not through protein-based recognition, like CRISPR-Cas systems, but via chemical specificity and strategic targeting through conjugation with DNA-recognizing molecules, such as synthetic oligonucleotides or triplex-forming oligonucleotides (TFOs) (Panattoni et al., 2020).

These artificial restriction enzymes are composed of programmable DNA-binding domains, such as the zinc-finger motifs or TALEs, fused to nucleases like FokI. By customizing the recognition domain, the ARCUT enzymes can be designed to target virtually any sequence in the genome. Following cleavage, the cell’s repair machinery, NHEJ or HDR, introduces genetic modifications at the break site. This mechanism underpins ARCUT’s utility for precise genome editing. ARCUT utilizes an artificial oligonucleotide-based recognition system, which enables precise targeting of DNA sequences while maintaining flexibility across different genetic backgrounds. Unlike protein-dependent editing tools, ARCUT operates without enzymatic components, reducing the likelihood of off-target effects and increasing its specificity in genome engineering (Katada and Komiyama, 2011) (Figure 1c). These enzymes are synthetic constructs designed to recognize unique DNA sequences and catalyze DSBs with high precision. ARCUT typically integrates a recognition domain and a cleavage domain. The recognition (binding) domain is engineered to bind to specific DNA sequences, while the cleavage domain induces DSBs at the target site. For improvements in genome-editing techniques, ARCUT is a potent alternative to conventional techniques available. The major advantage is that, it allows homologous recombination in polyploids, for example, wheat and canola, which are otherwise difficult for genome editing because they possess homoeologous gene copies combined with a very high degree of complexity of the genome. Since, ARCUT provides more specific targeting, this translates to more consistent edited attributes, thereby offering high utility in crop improvement and plant breeding programs. Moreover, there seems to be a regulatory advantage for ARCUT as it does not introduce foreign DNA into the plant systems and may not face some restrictions against GMOs in some parts of the world, thus possibly leading to quicker acceptance of the technology by the commercial agricultural sector.

Besides targeted gene editing, ARCUT has a potential to modulate epigenetic changes and induce gene silencing. Some studies seem to show that DNA/RNA hybrids, involved in transcriptional regulation, can be selectively sliced by ARCUT (Shaw and Arya, 2008; Futai et al., 2016). This, in turn, makes it a handy tool for looking at gene expression in plants and even for creating stress-resistant crops with better yield. On the downside, ARCUT’s performance in GC-rich regions still poses a challenge, and its specificity can get thrown off in high salt conditions often found in plant cells (Komiyama, 2014). ARCUT utilizes PNAs which can be less stable and efficient with poor water solubility in cellular environment compared to conventional DNA cutting tools, especially under physiological conditions. Furthermore, in order for ARCUT to actually work inside living plants, it needs to be delivered into plant cells at the desired genomic regions. Through further optimization of delivery methods or by coupling ARCUT with delivery systems that really integrate with plant systems, its utility in plant biotechnology may scale much higher.

ARCUT may be preferable to CRISPR-Cas9 in several specific scenarios where precision, reduced off-target effects, and cleaner repair outcomes are essential. CRISPR is more widely used due to its ease and versatility. ARCUT’s chemical-based mechanism offers unique advantages in targeted applications when high-fidelity homologous recombination is required, during protein-free editing systems, or targeting repetitive or structurally constrained regions. Overall, ARCUT comes off as a decent groundbreaking way to edit genomes, holding lots of promise for plant engineering. Its precision and non-reliance on proteins and no requirement of regulatory checks, makes it a tempting alternative to other tools like CRISPR-Cas and ZFNs. As further research fine-tunes its performance and sorts out delivery challenges, ARCUT could very well revolutionize how we approach genome editing in plants for better crop improvement.

### Single-stranded Oligonucleotide Annealing-mediated Targeted Integration (SATI)

SATI utilizes single-stranded oligodeoxynucleotides (ssODNs) as templates to guide the targeted integration or correction of specific genetic sequences and enables precise genetic corrections without causing DSBs (Suzuki et al., 2019). Initially, λ-Red recombination-based system was used in *Escherichia coli*, indicating that ssODNs-mediated gene repair involves the process of DNA replication (Huen et al., 2006). SATI employs single-stranded DNA annealing, where the alignment of ssODNs with complementary sequences in the target DNA, facilitate the precise insertion or replacement of genetic material (Figure 1d). SATI achieves strand-specific annealing of ssODNs through a combination of natural DNA repair processes and strategic oligonucleotide design, despite lacking DSBs. Unlike CRISPR-Cas9, which relies on DSBs to trigger repair machinery, SATI utilizes nicking or endogenous DNA repair pathways to enable precise sequence integration. By avoiding DSBs, SATI minimizes the risks of off-target effects and genomic instability that often accompany traditional genome editing tools. The non-invasive method of genome modification is particularly effective for editing crucial genomic areas where DSBs would result in harmful effects. The use of ssODNs in SATI eliminates the requirement for HDR that remains inactive in non-dividing cells, thus, enabling its application across various organisms and developmental stages (Suzuki et al., 2019). Moreover, ssODNs are relatively simple to design and synthesize, making SATI a cost-effective alternative to protein-based genome editing systems.

The principal use of SATI technology exists in therapeutic genome editing where it targets the correction of genetic disorders stemming from point mutations or small deletions. The SATI technique shows potential for treating Hutchinson-Gilford Progeria Syndrome (HGPS) by accurately correcting mutations while minimizing genomic disruption (Koblan et al., 2021).

In an early application of this technology in plants, oligonucleotides were used to target genes such as the acetolactate synthase (*ALS*) gene in tobacco (Beetham et al., 1999) and other genes in maize (Zhu et al., 1999) and oilseed rape (Gocal et al., 2015). In agricultural biotechnology, precision matters, SATI minimizes unintended mutations while maximizing the accuracy of genetic modifications. Chromosomal rearrangements and unexpected genomic changes become less probable when DSBs are eliminated. SATI provides enhanced safety for precise genetic modifications when editing regulatory regions and coding sequences in plant and animal genomes. SATI holds significant promise for editing polyploid plant genomes with greater precision than CRISPR, particularly in applications, where minimizing off-target effects, avoiding DSBs and achieving allele-specific edits are critical. Polyploid crops such as wheat, cotton, sugarcane contain multiple copies of each gene. CRISPR-induced DSBs can cause random insertions or deletions across homeologs, leading to unintended or unpredictable edits. SATI avoids DSBs entirely and uses strand-specific ssODNs with homology arms, allowing for allele-specific targeting with minimal collateral damage. In polyploid species, precise editing of one or a few homeologs without affecting others is often desired. SATI can enable homoeolog-specific editing using well-designed mismatches in the ssODN.

SATI is considered a safer genome editing method in terms of genome integrity because it bypasses the use of DSBs which are the primary source of chromosomal rearrangements in editing tools like CRISPR-Cas9. Chromosomal rearrangements such as translocations, inversions, and large deletions are commonly triggered by erroneous repair of DSBs via NHEJ or HDR. Since SATI does not rely on DSBs, it inherently eliminates misjoining of distant DNA ends, inter-chromosomal recombination, and large-scale genomic deletions. By leveraging ssODNs to guide the edit via base-pairing, SATI bypasses the genome’s emergency repair pathways, which are often error-prone. The homology-directed annealing between the ssODN and the complementary DNA strand ensures that the repair is localized and specific. While hurdles persist, continuous advancements aim to reveal SATI’s full capabilities and establish it as an essential technology to tackle genetic disorders and agricultural improvements.

## Prokaryotic Argonautes-based editing

### Short Prokaryotic Argonaute, DNase and RNase-Associated (SPARDA)

Apart from CRISPR-Cas, pAgos (prokaryotic Argonautes) are the second defence system for prokaryotes (native to bacteria and archaea), which detect and cleave the nucleic acid with the help of guide oligonucleotide, complementary to the target DNA (16–21 nt) (Agapov et al., 2024). Argonautes are programmable endonucleases found in all the eukaryotic and prokaryotic organisms (Ryazansky et al., 2018). In contrast to the eukaryotic AGOs, that mediate the RNA silencing using RNA guide, the pAgos targets the RNA as well as DNA using RNA or DNA guide (often phosphorylated or hydroxylated at the 5′ end) (Ryazansky et al., 2018). The pairing of the bound guide to its complementary sequence activates the endonuclease activity of pAgo, which then cleaves the phosphodiester backbone of the substrate (Graver et al., 2024). There are two different types of pAgo, varying in their architecture, long pAgo (active or inactive) and short pAgo. The long pAgos contain six domains, N-terminal, L1, PIWI/Argonaute/Zwille (PAZ) (anchors to the 3′ end of guide), L2, MID (middle domain) (binding pocket for 5′ end of guides), and the PIWI (cleavage activity) (Wang et al., 2024).

**FIGURE 2:**
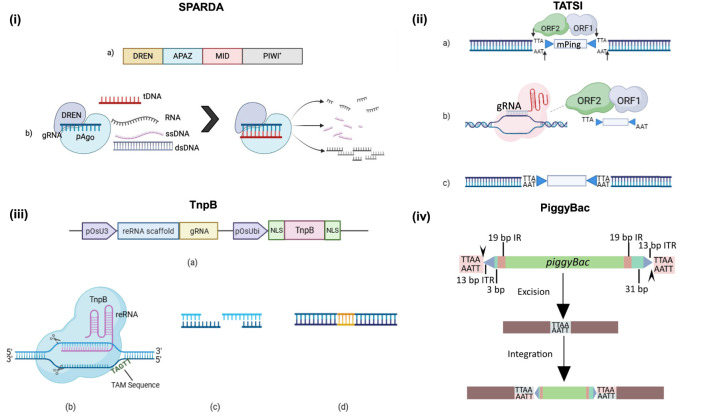
(i) (a) Schematic architecture of short pAgo (asterisk * indicates inactive domain). The long pAgos contain domains including the effector nuclease domain, DREN, PIWI/Argonaute/Zwille (PAZ), MID (middle domain) (binding pocket for 5′ end of guides), and the PIWI (for cleavage activity). (b) SPARDA-mediated degradation of nucleic acids. DREN is activated only upon the binding of gRNA to the target DNA degrading a wide spectrum of nucleic acids in the presence of divalent cation, Mg^2+^ or Mn^2+^. (ii) Schematic diagram showing TATSI-mediated targeted gene insertion: (a) The ORF1 and ORF2 (fused or unfused) excise the *mPing* producing the staggered cuts at both the ends, the light blue box represents *mPing*. (b) The gRNA localizes the cargo to the target site and induces a break in the sequence. (c) mPing inserts into the site introducing the TTA or TAA in the sequence. (iii) (a) The general structure of construct for TnpB-based editing. (b,c,d) represents the sequential steps of technique, i.e., binding of gRNA and nuclease loading, dsDNA break and indel mutations after repair mechanism. TnpB forms an RNP complex with right end (re) RNA and recognizes the 5′-TTGAT (TAM) to initiate a double strand staggered cut in the DNA. (iv) Illustration showing the excision and integration of piggyBac transposon in the TTAA element. IR, Inverted repeats, ITR, Inverted terminal repeats. This transposon integrates at the TTAA site and duplicates the TTAA element on the edges of ITRs during the integration leaving no footprints in the genome.

The short pAgos are truncated, retaining MID, and having inactive PIWI (lacking DEDX tetrad) with probable fusion of analogous PAZ (APAZ) domain, that is usually linked to the effector nucleases (sirtuin domain 2 (SIR2), SPARSA, or Toll-interleukin-1 receptor (TIR), SPARTA homology domain (Wang et al., 2024) (Figure 2i). This architecture suggests the modular activity of the short pAgos, with MID-PIWI domain involved in guided target binding, followed by the target cleavage via associated nucleases. Unlike CRISPR-Cas, the pAgos may result in either staggered or blunt ends. The SPARSA and SPARTA system involves the cell death via the hydrolysis of NAD+, as both of the effector nucleases are the NADases. A research group from Russia discussed a new short pAgo system, SPARDA (short prokaryotic Argonaute, DNase and RNase associated) having a DREN coupled with an effector nuclease, DUF4365, whose key catalytic residues and the predicted active site align with TnsA transposase from the Tn7 family, indicating towards its nuclease activity (Prostova et al., 2024). SPARDA is a bi-partite immune system consisting short pAgo protein and co-encoded nuclease, with indiscriminate cleavage activity. The study shows that the effector nuclease domain, DREN, is activated only upon the binding of gRNA to the target DNA (RNA guided DNA recognition), degrading a wide spectrum of nucleic acid in the presence of divalent cation, Mg^2+^ or Mn^2+^. Attributed to its higher flexibility, guide stability, and compact cargo size, this technique has the potential to open a new field of biotechnology.

As SPARDA is a recently identified system and in exploratory phase, further experimental validation and functional characterization is required to assess its potential as a genome editing tool in plants.

## Transposon-mediated genome editing

### Transposase-assisted Target-site Integration (TATSI)

TATSI is a novel genetic integration method that utilizes the natural capacity of transposases to mediate targeted integration of genetic elements. Transposases are enzymes that facilitate the excision and insertion of transposable elements (TE), or more fondly referred to as “jumping genes,” from location to location in the DNA. Transposons, are self-transposable genetic elements that can independently excise themselves and integrate into other genomic locations (Hayward and Gilbert, 2022). Adapted from natural transposons, this mechanism (TATSI) has been used for targeted and programmable genetic manipulation in many organisms (Tao et al., 2025). The engineered transposase at the heart of the TATSI system is often modified to increase activity or specificity, or to bind to target DNA sequences. Scientists achieve targeted transposase activity within the genome by bonding enzymes with DNA-binding structures like ZFNs along with TALEs and Cas proteins. Programmability allows scientists to integrate genetic elements at specific sites within the genome addressing the issue of random insertions and essential gene disruption seen with previous methods (Liu et al., 2024).

Recently, Liu et al. (2024), has introduced a novel groundbreaking version of CRISPR that pairs the function of CRISPR-Cas9 with the rice transposon system *Pong*. This system enables sequence-specific targeting of concerned DNA in plant genomes. The *Pong* transposase consists of two open reading frames (ORFs), ORF1 and ORF2, essential for the excision and integration of the non-autonomous 430-bp *mPing* element, flanked by TTA or TAA repeats (Figure 2ii). In this research, the ORF1 and ORF2 were introduced to the system, in fused or unfused state with the Cas9, nickase and deactivated Cas9 protein, in different configurations, totalling to twelve fusion proteins. The results suggest that the successful site-specific integration requires the presence of ORF1, ORF2 and active Cas9. The unfused Cas9 with ORF2 configuration demonstrated higher rates of *mPing* excision (98.7%), and targeted insertion (35.5%) as compared to that of a fused configuration with 75.0% *mPing* excision and 6.7% targeted insertion (Liu et al., 2024). This difference may be due to the transposase proteins’ property to bind to the *mPing* element on either side, when extrachromosomal, which cannot be achieved optimally in fused configuration.

To validate the applicability of this technique in the crop plant, it was applied to soybean, where it yielded positive results (Johnson et al., 2021). Hence, TATSI has the potential to work in any transformable crop genome, except for rice, in which it is likely to be epigenetically silenced. This technique represents a novel strategy to precisely integrate the transgene, in efficient and site-specific manner. Since, the technique was shown to function in both the model plant and crop plant, it can be considered a critical advancement in the field of crop biotechnology. It also allows multiplexing, that is, multiple gRNAs can be used in a single transformation event, with stable trait integration. Despite being capable of inserting the DNA in site specific manner, the orientation specific integration and free transpositions still pose a challenge in the technique. Additionally, the efficiency of integration depends upon the chromatin state that can also limit the range of usable sites. These challenges may be addressed in future making it a scalable strategy.

### PiggyBac transposon-mediated editing

Along with TATSI, *piggyBac*, derived from cabbage looper moth (*Trichoplusia ni*), is another transposon system which operates in a scarless manner. The transposon contains the transposase, PBase, with inverted repeats, including, 13-bp inverted terminal repeats and a 19-bp sub-terminal inverted repeat. This transposon integrates at the TTAA site and duplicates the TTAA element on the edges of ITRs during the integration, leaving no footprints in the genome (Li et al., 2013) (Figure 2iv). A binary system has been adopted to use it in genetic engineering. It includes the PBase and the ITR element containing the cargo. To validate the activity of this system in plants, Nishizawa-Yokoi *et al* used homology directed gene targeting in rice using positive-negative selection and they found positive result for the function of insect *piggyBac* transposase (ePBase) as well as hyperactive PBase (hyPBase) (Nishizawa-Yokoi and Toki, 2023). They have also paired the system with the CRISPR-Cas9, as the system allows a larger cargo size. They transformed the rice calli with CRISPR-Cas9 construct inserted in the *piggyBac* transposon. Followed by the expression of Cas9 and gRNA, the mutation is induced in the target sequence. The transient expression of PBase in the next step leads to the removal of CRISPR-cassette from the genome leaving the genome free from the cassette footprint. They used two types of hyPBase, one codon optimized for mouse (hyPBase) and another optimized for rice (OshyPBase) itself. The results showed the lower excision frequency (40%–50%) in this system as compared to that of gene targeting approach, i.e., replacing a gene via homologous recombination (>90%) with no significant difference in results of different PBase. This variation is likely due to the different strategies for insertion, the chromosomal position effect of insertion and DNA methylation state (Kitamura et al., 2001). The transposition of piggyBac has been demonstrated in a dicot plant, *Nicotiana tabacum,* where two transgenic lines, one with transposable element and one with transposase, were crossed together to validate the activity. The transposition frequency in progeny was found to be very low, i.e., 2%, that can be due to the utilization of wild type PBase, different experimental approach, or the plant physiology (Johnson and Dowd, 2014). This technique provides an elegant solution for precise genome editing. The piggyBac insertion coupled with CRISPR-Cas technique overcomes the problem of removing transgene from genome edited crops. The transient expression of hyPBase eliminates the CRISPR construct leaving behind the edit only. This technique presents a broad scope of potential improvements and advancements, in terms of efficiency in different systems.

### Tiny TnpB: compact gene scissors

The Cas9 and Cas12a nucleases are suggested to be evolved from the transposon nucleases, IscB and TnpB, respectively. One of the main challenges associated with CRISPR-Cas9 technique is its large cargo size which hinders its delivery and insertion efficiency. To address this problem, scientists discovered a miniature and effective alternative to CRISPR-Cas9, named TnpB, which is a protein of ∼400 aa, much smaller than Cas9 (∼1000–1400 aa) and Cas12a (∼1300 aa). It is a part of the Obligate Mobile Element Guided Activity (OMEGA) RNA-guided nuclease family. The mobile genetic elements (MGE) family (IS200/IS605 and IS607) to which TnpB belongs, consists of subterminal palindromic elements on the left end (le) and right end (re). The TnpB is an accessory gene of MGE, which forms an RNP complex with right end RNA and recognizes the 5′-TTGAT to initiate a double strand staggered cut in the DNA. As the latter is analogous to the PAM sequence, it is named as transposon-associated motif (TAM) (Figure 2iii). Despite the shared characteristics between TnpB and Cas12a, they differ in their off-target efficiency, as TnpB recognizes shorter RNA-target DNA duplex (12 bp) and Cas12 recognizes ∼20 bp duplex, thereby offering lower off-target risk to the latter (Omura et al., 2023). Cas12 system also provides a greater number of compatible target sites due the flexible PAM sequence (TTTV), while TnpB with narrower or more restrictive TAM, limits the number of sites (Zhou et al., 2022; Karvelis et al., 2021) The TnpB is basically a homing nuclease, helping the organism to retain the MGE, if its integration fails.

Karmakar et al. (2024), and Li et al. (2024), have developed and optimized a compact genome editor based on TnpB to achieve efficient editing in *Oryza sativa*. The former group cloned the TnpB gene under the OsUbi10 promoter and the right end element (reRNA) under the OsU3 promoter, while the latter used the OsU6a promoter for reRNA and the same promoter for TnpB. The optimization of the TnpB nuclease system enabled Indian and Chinese scientists to achieve editing efficiencies of 33.58% and 100% (11/11), respectively (Karmakar et al. 2024; Li et al. 2024) (Figure 2iii). In Arabidopsis, researchers analyzed T1 generation of *A. tumefaciens*-mediated transformed plant and got 14% mutation efficiency for TnpB-mediated genome editing. The extension of this study to medicinal plants, *A. annua, S. miltiorrhiza, S. baicalensis, I. indigotica,* and *C. pilosula* yielded positive results with mutations in the target region (Lv et al., 2024). TnpB has proven effective in both monocots and dicots; future efforts can further optimize it by focusing on appropriate promoters, relaxing TAM requirements, codon optimization, and engineering enzyme variants to improve efficiency and creating a more versatile genome editor.

## Helicase-mediated genome editing

### Helicase-assisted Continuous Editing (HACE)

**FIGURE 3:**
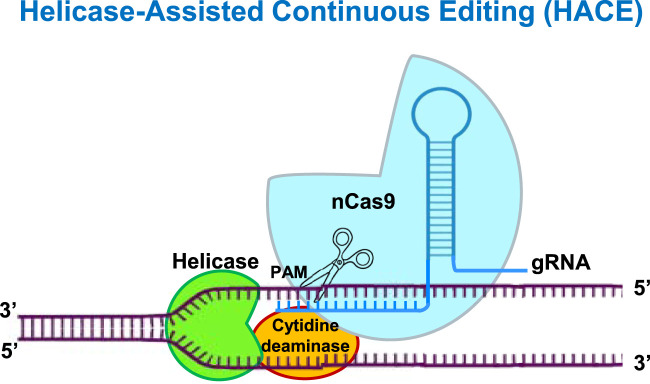
Helicase-Assisted Continuous Editing (HACE) involves helicase enzymes in regular CRISPR-Cas system, which leverages the precise gene editing at specific sites without affecting rest of the genome. To target it to a specific genomic location, the nicking variant of Cas9 (nCas9) was used in the system, deploying the fusion of cytidine deaminase and a helicase. The nCas9 creates a nick at a gRNA-guided location, the helicase starts DNA unwinding from the nick site, and generate random mutations in the process with the help of cytidine deaminase.

Helicase-Assisted Continuous Editing (HACE) represents a groundbreaking progress in blending the property of helicases of unwinding the DNA to enhance the efficiency of genome editing. Helicases, are capable of unwinding double-stranded DNA and facilitating several crucial cellular processes such as DNA replication, repair and transcription. In the HACE system, helicases are exploited to overcome structural barriers like chromatin compaction and stem-loops, enhancing accessibility of target genomic regions for editing machinery. This activity converts the highly complex and inaccessible DNA for modifications involving cleavage and editing at specific DNA sites (Singleton et al., 2007; Chen et al., 2024) (Figure 3). The HACE mechanism integrates helicases with classical genome-editing tools, such as CRISPR-Cas systems, ZFNs, and TALENs. Together with the editing tools, the helicases are guided to specific locations through gRNAs as in the CRISPR systems or engineered DNA-binding domains as in ZFNs and TALENs, leading to site-specific unwinding and enhanced editing. Such capabilities make HACE an invaluable tool in studies of gene regulation and synthetic biology constructs.

HACE Editor (HE) deploys a fusion of a helicase and deaminase enzyme to generate multiple somatic mutations across an extended genomic region. To target it to a specific genomic location, the nicking variant of Cas9 (nCas9) was used in the system, deploying the fusion of cytidine deaminase and *Geobacillus stearothermophilus* PcrA helicase. A uracil DNA-glycosylase inhibitor (UGI), which has been shown to facilitate C:G to T:A conversion, was also attached to it. The nCas9 creates a nick at a gRNA-guided location, the HE starts DNA unwinding from the nick site, and generate random mutations in the process with the help of cytidine deaminase (Chen et al., 2024). HACE avoids the need of multiplexing sgRNAs for mutations across several sites as a large genomic region can be mutated using a single sgRNA with the help of a helicase and a deaminase. HACE demonstrates wide applicability because it temporarily alters regulatory elements through modifications which do not result in lasting changes (Chen et al., 2024). HACE provides genome editing capabilities in intricate DNA regions along with adaptable integration with available genome editing frameworks that increases its functionality throughout multiple biological systems, both in research laboratories and therapeutic practices (Chen et al., 2024).

The CRISPR-associated hurdles of off-target effects and delivery to various plant systems remain with HACE too. The widespread usage of HACE confronts conventional issues stemming from its unintentional helicase reaction in other regions of DNA. Engineering of helicases for better processivity and fidelity, and using base-editing enzymes along with cytidine and adenosine deaminases, can further improve the efficacy of the system. New advancements seek to enhance helicase targeting precision through improved mechanisms that reduce non-targeted edits. The potential of HACE technology will expand for research and clinical applications with its combination to base editing and prime editing technologies. With time, the technique can be applied for genome editing in plants as well.

### Type IV-A CRISPR system

**FIGURE 4:**
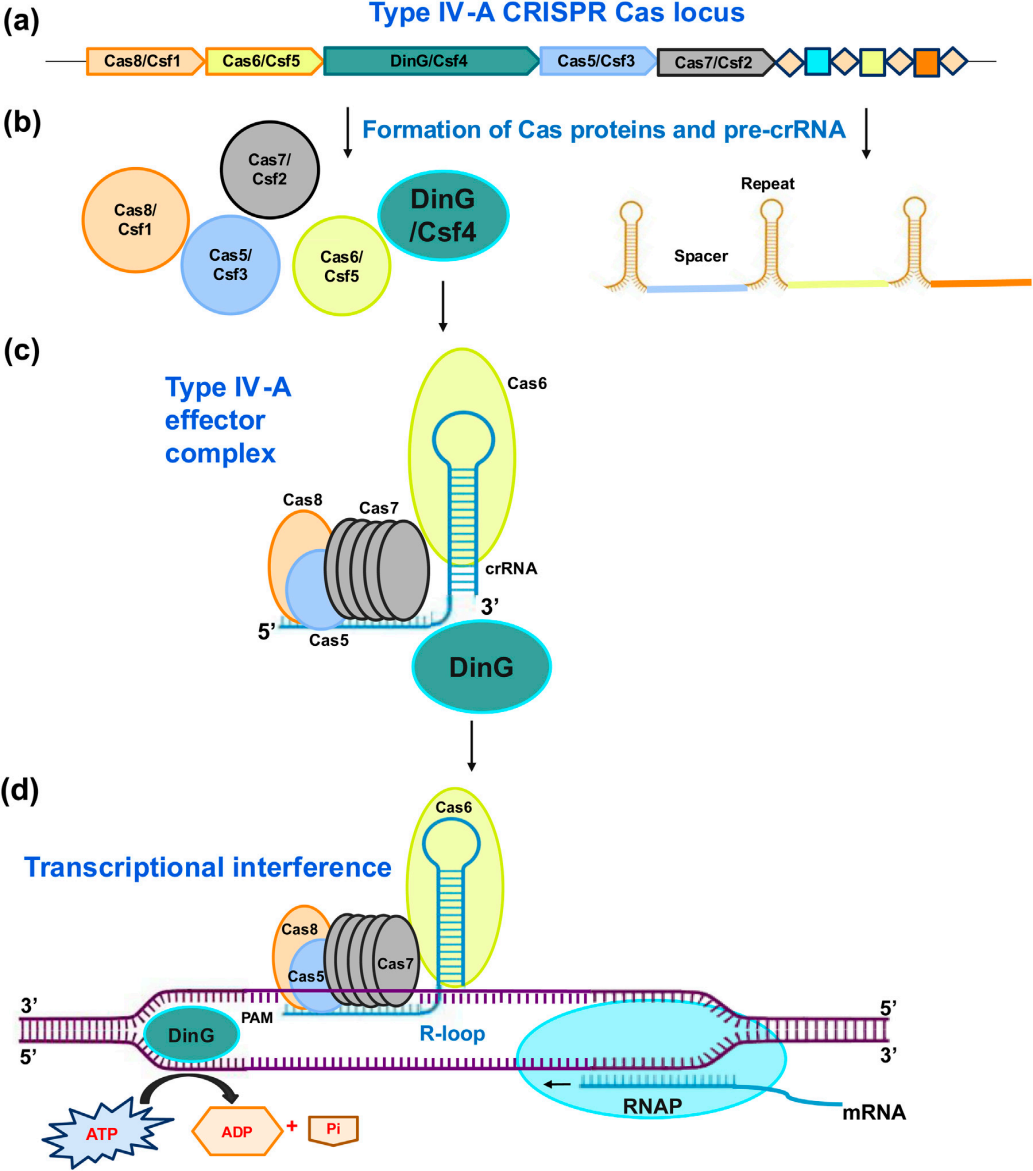
Illustration presenting the functional process of a CRISPR-Cas type IV-A locus via transcriptional interference. **(a)** Components of CRISPR-Cas type IV-A locus **(b)** Translation of various effector subunits and transcription of CRISPR-array precursor CRISPR RNA (pre-crRNA). **(c)** Processing of pre-crRNA into mature gRNA by Cas6 and recruitment of Cas proteins- Cas5, Cas8 and various Cas7 subunits making an effector complex. **(d)** Recognition of target DNA sequence and transcriptional interference by DinG endonuclease in an ATP-dependent manner. Different effector subunits are presented by different color codes. Cas6/Csf5 endoribonucleases proceed the transcription process of CRISPR-array into pre-crRNA and then to mature crRNA which further brings the Cas proteins- Cas8/Csf1, Cas5/Csf3 and various subunits of Cas7/Csf2 to form a crescent shaped effector complex which identifies a short PAM in the upstream region of crRNA complementary sequence, recognizing precise target. The ATP-dependent action facilitated by DinG helicase promotes effective transcriptional interference following the DNA target interaction. This interference action lowers gene expression in case the knock-outs of target genes jeopardizes viability.

The Type IV-A CRISPR system introduces a dynamic approach to achieve gene silencing (CRISPR interference or CRISPRi) without inducing permanent changes in the DNA sequence, making it a reversible and precise tool for controlling gene activity, particularly where transient gene silencing is required (Cepaite et al., 2024). Type IV-A system encodes three core type IV genes (*csf1, csf2*, and *csf3*), an endoribonuclease (*cas6*/*csf5*), a CRISPR array, and a putative helicase (*dinG*) (Taylor et al., 2019, 2021). Type IV CRISPR–Cas system using crRNA-guided effector complexes, has been studied in *Aromatoleum aromaticum* (Ozcan et al., 2019). Guide RNAs direct the effector complex to specific sequences in the genome, precisely targeting the sequence where gene silencing is desired. The effector complex then recruits DinG, which unwinds the DNA double helix in the targeted region (Domgaard et al., 2023). This unwinding creates a R-loop in the structure of DNA that prevents transcription, without causing DSBs or permanent changes to the genome. This silencing can be fully abolished immediately, when the intervention is withdrawn, and provide unique flexibility and control of gene expression (Guo et al., 2022; Cepaite et al., 2024; Cui et al., 2023) (Figure 4).

Type IV-A CRISPR system is capable of transient gene silencing under controlled conditions. This enables researchers to study dynamic biological processes and gene function, reversibly (Cui et al., 2023). Moreover, the system can be used to fine-tune the level of gene activity without modifying the underlying DNA sequence, making it ideal for use in experiments that depend on tight regulation of gene activity (Sanchez-Londono et al., 2024). Moreover, the use of the Type IV-A system avoids DSBs and off-target effects, increasing the safety of this system, particularly in therapeutic applications (Cepaite et al., 2024; Cui et al., 2023). The system has a potential to be used for plant genome editing that has not been explored so far. Low editing efficiency and off-target effects remain a concern for Type IV-A CRISPR system and warrants further improvement.

## Conclusion and future perspectives

While genome editing tools, particularly CRISPR-Cas present significant opportunities for agriculture, several challenges including, but not limited to off-target effects, regeneration capability, delivery efficiency, editing efficacy and tissue-specific editing still need to be addressed to achieve the full potential. There are reports of successful utilization of CRISPR-Cas technology to enhance various agricultural traits such as nutrition, pathogen defence, yield and abiotic stress tolerance. The CRISPR-Cas system has expanded and Cas variants, base editors and prime editors hold promise in further refining plant genome engineering. The problems with CRISPR, related to off-target effects, delivery, and precise gene or RNA editing can be overcome using non-CRISPR methods. More recently, the expanding suite of next-generation technologies including Type IV-A CRISPR, LEAPER, SATI, RESTORE, RESCUE, ARCUT, SPARDA and transposon-based approaches like TATSI and piggyBac introduce novel opportunities that merit deeper exploration. These emerging tools offer varied mechanisms such as DSB-free editing with SATI, RNA-specific modifications with LEAPER, RESTORE and RESCUE, and helicase-assisted access with HACE and gene-silencing through Type IV-A CRISPR system, offering precise transgene insertions with minimal genomic disruption. Their development reflects a shift towards more nuanced and customizable genome interventions with applications ranging from transient gene expression modulation to stable trait enhancement.

TATSI and piggyBac offer a significant advantage over conventional integration approaches as they avoid low frequency and random insertion with unintended mutations in the genome. Notably, piggyBac enables footprint-free transposition ensuring cleaner edits. RESTORE and LEAPER utilize endogenous ADAR enzyme and create transient RNA modifications. The LEAPER system utilizes engineered RNA oligonucleotides complementary to the target sequence, whereas the RESTORE system utilizes a chemically synthesized ASO to catalyse the A-to-I conversion in the presence or absence of the catalyst Cas13 protein. These allow transient changes in the RNA and do not warrant regulatory impositions. SATI utilizes ssODNs to enable precise genetic changes without causing DSBs, thereby reducing the risk of unintended changes in the genome. SATI marks a major progress within genome editing techniques by delivering a dependable and flexible instrument for precise genetic alterations. The helicase-based systems provide enhanced access to target genomic regions for better editing efficiency. Among SPARDA, TnpB and piggyBac, SPARDA is currently in the infancy stage, i.e., it requires more detailed and focused research to be applied. The coupling of TnpB, aka, compact scissors and piggyBac transposon system with large cargo capacity can be a compelling approach for genome editing in plants, since they both have been validated in plants.

Minimizing off-target effects, increasing delivery efficiency, and tissue-specific editing remain the focus in genome engineering. Delivery of arRNA, ASOs or large constructs into recalcitrant plant tissues remains major bottleneck, especially in monocots with complex transformation requirements. Species-specific differences in ADAR expression, chromatin accessibility, or DNA repair pathways may further influence editing efficiency and specificity. Additionally, limited data exists on off-target effects, epigenetic consequences or phenotypic stability over generations in edited plants using these newer tools. Therefore, a systematic benchmarking of these tools in both model and non-model species alongside optimization of delivery platforms (e.g., nanoparticles, viral vectors, electroporation) is urgently needed. Furthermore, the potential ecological regulatory and societal implications especially in food crops must be carefully evaluated before widespread adoption.

Despite these hurdles, the continuous evolution of genome editing technologies is reshaping the future of agricultural biotechnology. By exploiting advancements in editing tools, food security is enhanced through sustainable agricultural production. Future research should focus on optimizing these technologies for broader agricultural applications while addressing ethical, regulatory, and societal considerations. In conclusion, while next-generation genome editing platforms mark a significant leap beyond CRISPR-Cas, their full potential can only be harnessed through integrated research that addresses both technical optimization and translational scalability. A more nuanced understanding of their biological mechanisms, delivery strategies and context-specific applications will be crucial in steering their deployment for sustainable and equitable agricultural transformation.
